# Correction: Kim et al. The Medium Cut-Off Membrane Does Not Lower Protein-Bound Uremic Toxins. *Toxins* 2022, *14*, 779

**DOI:** 10.3390/toxins15010064

**Published:** 2023-01-11

**Authors:** Yang Gyun Kim, Sang Ho Lee, Su Woong Jung, Gun Tae Jung, Hyun Ji Lim, Kwang Pyo Kim, Young-Il Jo, KyuBok Jin, Ju Young Moon

**Affiliations:** 1 Division of Nephrology, Department of Internal Medicine, Kyung Hee University School of Medicine, Seoul 05278, Republic of Korea; 2 Department of Biomedical Science and Technology, Kyung Hee Medical Science Research Institute, Kyung Hee University, Seoul 02453, Republic of Korea; 3 Department of Applied Chemistry, Institute of Natural Science, Global Center for Pharmaceutical Ingredient Materials, Kyung Hee University, Yongin 17104, Republic of Korea; 4 Division of Nephrology, Department of Internal Medicine, Konkuk University Medical Center, Seoul 05029, Republic of Korea; 5 Division of Nephrology, Department of Internal Medicine, Keimyung University Dongsang Hospital, Keimyung University Kidney Institute, Daegu 42601, Republic of Korea

## Error in Table

In the original publication [1], there was a mistake in Table 2 as published. The information for dialyzers including inner diameter, wall thickness, effective surface area, clearance of inulin, and UF coefficient was incorrect. The corrected Table 2 appears below. The authors state that the scientific conclusions are unaffected. This correction was approved by the Academic Editor. The original publication has also been updated.

## Figures and Tables

**Table 2:** The characteristics of the dialyzers.

	HF-HD	Post-OL-HDF	MCO-HD
Dialyzer	Fx CorDiax 80	Fx CorDiax 800	Theranova 400
Inner diameter (μm)	185	210	180
Wall thickness (μm)	35	35	35
Membrane polymer	polysulphone-PVP blend	polysulphone-PVP blend	polyarylethersulphone-PVP blend
Effective surface area (m^2^)	1.8	2.0	1.7
Sieving coefficients of albumin	<0.001	<0.001	0.008
Clearance of inulin	135	178	183
UF coefficient (mL/h/mmHg)	64	62	48

PVP, polyvinylpyrrolidone; UF, ultrafiltration.
